# The Cytochrome CYP4 in Breast and Other Cancers

**DOI:** 10.3390/biology14070812

**Published:** 2025-07-04

**Authors:** Gloria M. Calaf, Leodan A. Crispin, Felipe Ossandon-Acosta, Summer Perez-Tapia, Luis N. Ardiles

**Affiliations:** 1 Instituto de Alta Investigación, Universidad de Tarapacá, Arica 1000000, Chile; kcrispi@gestion.uta.cl (L.A.C.); lardilesa@academicos.uta.cl (L.N.A.); 2 Departamento de Química, Facultad de Ciencias, Universidad de Tarapacá, Arica 1000007, Chile; felipe.ossandon.acosta@alumnos.uta.cl (F.O.-A.); summer.perez.tapia@alumnos.uta.cl (S.P.-T.)

**Keywords:** breast cancer patients, cytochrome P450 superfamily, bioinformatics analysis

## Abstract

Breast cancer has emerged as the leading cause of death among females worldwide. The CYPs play a crucial role in carcinogenesis. The role of the CYP enzyme family, particularly the CYP4 family, in cancer biology has attracted significant attention in recent years. Bioinformatics indicated that breast cancer is influenced by genes like *CYP4B1*, *CYP4F12*, and *CYP4F3*. *CYP4B1* has a non-significant correlation with *BRCA1* and *BRCA2*, but a positive correlation with *ESR1* in the basal subtype. *CYP4F12* has a significant positive correlation with *BRCA1* in the Luminal B subtype, but not with *BRCA2,* and a positive correlation with *ESR1* in the basal subtype. *CYP4F3* has a significant positive correlation with *BRCA1* in the Luminal A and Luminal B subtypes and with *BRCA2* in Her2, Luminal A, and Luminal B subtypes, and a positive correlation with *ESR1* in the basal subtype and Luminal B patients. This article aims to emphasize the functional importance of CYP4, highlighting the complex interplay between CYP enzymes and estrogen receptors in breast cancer, and indicating new avenues for future research and potential therapeutic interventions. In addition, their expression profiles and alterations were examined across various organs and cancer types. These findings underscore the potential relevance of these genes as predictive biomarkers and prospective therapeutic targets in specific cancer settings.

## Simple Summary

The complex relationship between CYP enzymes and estrogen receptors in breast cancer is highlighted, revealing potential therapeutic interventions. The study reveals genomic alterations and differential expression patterns between normal and tumor tissues, suggesting a functional role in tumorigenesis. The positive correlation of these genes with estrogen receptor status strengthens their potential as predictive biomarkers.

## 1. Introduction

Cancer, a multifaceted group of diseases, is characterized by the uncontrolled growth and spread of abnormal cells, which can invade nearby tissue and metastasize to distant organs [1]. Breast cancer has become the leading cause of death in females worldwide, being responsible for 670,000 deaths in 2022, according to the World Health Organization [2]. The global incidence of cancer is increasing, driven by aging populations, lifestyle factors, and environmental exposures, placing an immense physical, emotional, and economic burden on society. These burdens are felt particularly in low- and middle-income countries, where healthcare systems often lack the resources for early diagnosis and access to treatment [3]. By contrast, in countries with robust healthcare infrastructures, cancer survival rates are improving thanks to advancements in early detection and prevention, and the availability of high-quality treatments [4].

Cancer types vary by gender and region. Among men, lung, prostate, colorectal, stomach, and liver cancers are the most prevalent, while women are more frequently diagnosed with breast, colorectal, lung, cervical, and thyroid cancers [5]. Despite the differences in cancer types and risk factors, certain biological mechanisms underlie the development and progression of tumors, and understanding these mechanisms is essential for developing new therapeutic strategies [6].

Estrogen receptor (ER), progesterone receptor (PGR), and erb-b2 receptor tyrosine kinase 2 (ERBB2) are three important receptors that are commonly utilized to categorize breast cancers. The lack of these receptors is a characteristic of TNBC, a subtype of breast cancer [7]. When the ER is positive, estrogens cause cancer cells to proliferate. However, TNBC does not have this receptor. TNBC also lacks PGRs, which are associated with the hormone progesterone. TNBC is more aggressive than other subtypes of breast cancer and is associated with a higher risk of recurrence and a worse prognosis [8]. African American women and young patients are the main victims of basal-like tumors, which account for 15% of all breast cancers [9]. Because TNBC does not react to hormone therapies or drugs that target HER2, therapeutic choices are more limited [10]. Standard treatment often includes chemotherapy, but researchers are also looking into immunotherapies and tailored medicines. Researchers are looking into the molecular characteristics of TNBC to identify potential targeted medications and biomarkers that could indicate how therapy will proceed. Clinical trials and innovative immunotherapy strategies have enormous potential to improve TNBC patients’ prognosis [11].

Many research studies have used microarray analysis to propose a new taxonomy of breast cancer based on genetic characteristics. Gene expression microarray-based class discovery studies have revealed at least five molecular breast cancer subtypes: basal-like, Her2, normal breast-like, luminal A, and luminal B [12,13,14,15,16,17]. The greatest distinction between the transcriptomes of estrogen receptor-positive (ERþ) and ER-negative (ER) breast cancers was found by microarray research. The basal-like group is a subject of debate regarding the molecular subtypes of breast cancer identified by gene expression profiling studies. The characteristics of basal-like breast cancer have been studied, and pathologists are aware of the distinctions between triple-negative and basal-like breast cancers [15,18,19,20].

The molecular subtypes identified by microarrays and immune histochemical markers are not often directly comparable [21,22]. The definition of basal-like breast cancers is not generally accepted. Using panels of immune histochemical markers and microarray-based expression profiling [23], several groups have tried to identify basal-like breast cancers. These include the following: (1) the triple-negative immune phenotype (absence of ER, PGR, and HER2 expression); (2) the expression of one or more high-molecular-weight/basal cytokeratins (CK5/6, CK14, and CK17); (3) absence of ER and HER2 expression in conjunction with CK5/6 and/or EGFR expression [22]; and (4) the absence of ER, PGR, and HER2 expression in conjunction with CK5/6 and/or EGFR expression. Histological characteristics [21,24], chemotherapeutic response [12,16,25,26,27,28,29,30,31], and distinct clinical presentations [32] are all displayed by these malignancies.

Estrogen receptor-negative (ER-) breast cancer is a heterogeneous disease that is characterized by an earlier time-to-lapse compared to ER+ breast tumors [33,34]. As opposed to estrogen receptor-positive (ER+) breast cancer, where the estrogen receptor signaling has a critical biological and therapeutic role, there is limited knowledge available regarding the pathophysiology of ER- disease. Therefore, to discover effective therapeutic strategies, there is a need for a better understanding of the biology of ER- breast cancer. This disease can be divided into molecular subgroups based on the expression microarray profiling, and the two most prominent ER- subgroups include molecular apocrine and basal subtypes [34,35,36]. The molecular apocrine subtype is characterized by a steroid response gene signature that includes the androgen receptor, FOXA1, TFF3, and a high frequency of ErbB2 overexpression [35,36,37].

An area of research that has attracted significant attention in recent years is the role of the cytochrome P450 (CYP) enzyme family, particularly the CYP4 family, in cancer biology. The cytochrome P450 enzymes are traditionally known for their function in drug metabolism and the biotransformation of endogenous compounds such as fatty acids and eicosanoids [38,39]. This is a large superfamily of integral membrane-conserved proteins present in animals, plants, and microorganisms [7].

These enzymes catalyze the oxidation of a wide range of substrates, including xenobiotics, and their activity is crucial for maintaining lipid homeostasis and detoxifying foreign substances [40]. However, emerging evidence suggests that the CYP4 family of enzymes may also have a profound impact on tumorigenesis, cancer progression, and the response to therapy [41]. The CYPs include exogenous (xenobiotics) and endogenous compounds, such as cholesterol, testosterone, progesterone, prostaglandin H2, corticosterone, retinoic acid, and integral membrane conserved proteins present in animals, plants, and microorganisms [42,43]. CYP is a subfamily of enzymes that catalyze the metabolism of drugs and other substances, such as estradiol, and also act in various metabolic processes in different cell tissue [7]. The CYP isoenzyme superfamily comprises 57 CYP genes and 58 pseudogenes, which are arranged into 18 families and 43 subfamilies in man [8]. These are heme-containing proteins that catalyze the oxidative metabolism of many structurally diverse drugs and chemicals [9].

In mammals, the CYP system is expressed in all examined tissue [42]. They predominate in the endoplasmic reticulum membrane and other cellular compartments, such as the cell surface and mitochondria [44]. The CYP superfamily is primarily located in the liver, small intestine, and kidney [45,46]. CYP enzymes catalyze different oxidation and some reduction reactions [47]. The CYP4A subfamily is responsible for encoding a variety of CYP enzymes. These enzymes can hydroxylate the terminal omega-carbon and, less commonly, the (omega-1) position on both saturated and unsaturated fatty acids. Additionally, they include enzymes that are involved in the omega-hydroxylation of different prostaglandins [48].

The present study aims to analyze the complex interplay between CYP enzymes and estrogen receptors in breast cancer, looking for new avenues for potential therapeutic interventions. However, this study emphasizes *CYP4B1*, *CYP4F12*, and *CYP4F3* gene expression levels, focusing on the tumor and normal tissue analysis; their expression, adjusted in stages; their correlation with *BRCA1*, *BRCA2,* and *ESR1*; the estrogen receptor status; and the overall survival analysis in breast cancer patients, while also exploring their expression profiles and alterations across other organs and cancer types.

## 2. Database Analysis

This review included datasets from web servers such as the Tumor Immune Estimation Resource v2.0 (TIMER2.0) [49] (http://timer.cistrome.org, accessed on 1 August 2024), the University of Alabama at Birmingham Cancer Data Analysis Portal (UALCAN) [50], (https://ualcan.path.uab.edu/, accessed on 12 November 2024), and the University of California, Santa Cruz, (UCSC) Xena functional genomics explorer [51] (https://xena.ucsc.edu/, accessed on 1 August 2024). Such datasets were used to explore *CYP4B1*, *CYP4F12*, and *CYP4F3* mRNA expressions in several types of cancer. Additionally, this exploration contained data from The Cancer Genome Atlas (TCGA) and The Genotype-Tissue Expression (GTEx) projects.

TIMER2.0 GeneDE module of the Cancer Exploration component provided the analysis of tumor and normal tissues, while statistical significance was computed via the Wilcoxon test, annotated with the number of stars (*: *p*-value < 0.05; **: *p*-value < 0.01; ***: *p*-value < 0.001). The gene expression levels of CYPs and breast cancer stages were retrieved from the UALCAN web server, and the significance difference was estimated by Student’s *t*-test, considering unequal variance. The correlation analysis was obtained using the Gene-Corr module of the Exploration component of TIMER2.0, and significance was facilitated by Spearman’s test. The University of California, Santa Cruz, UCSC Xena web server (https://xena.ucsc.edu/, accessed on 1 August 2024) provided online visualization of the estrogen receptor status of the genes under study, and the statistical significance was computed via a one-way ANOVA test.

The overall survival (OS) analysis was conducted using the Gene_Outcome module of TIMER2.0 [49]. This module used the Cox proportional hazard model modified by the clinical stage factor to provide the outcome significance. To display the normalized coefficient of the gene in the Cox model, a heatmap table was created. The Kaplan–Meier (KM) curves of the genes were obtained by clicking on a cell in the heatmap when surfing the web server. The statistical significance of the KM survival curves was assessed using the log-rank test. *p* < 0.05 was considered to indicate a statistically significant difference.

## 3. The Effect of CYPs on Several Types of Cancer

### 3.1. Breast Cancer

The CYP family consists of 11 subfamilies (CYP4A-CYP4M) that encode constitutive and inducible isozymes expressed in mammals, and CYP2E1 is one of the most active ROS-generating CYP isoforms [11]. Considering the link between oxidative stress and tumor growth, it has been hypothesized that CYP2E1-mediated ROS generation could regulate breast carcinogenesis [52]. Exogenous CYP substrates consist of polycyclic aromatic hydrocarbons and around 80% of the drugs in use today. Certain isoforms can activate pro-carcinogens, transforming them into actual carcinogens [53]. In addition, genetic variations in CYP enzymes can influence their catalytic activity, and these differences have been observed in various populations, linking them to a range of diseases and negative reactions to medications.

Authors [54] studied the expression of 21 cytochrome P450 (CYP) enzymes that were assessed in breast tumors. The results indicated that CYP4V2, CYP4X1, and CYP4Z1 expression were correlated with a higher tumor grade. Furthermore, the absence of CYP4V2, CYP2S1, CYP3A4, and CYP26A1 was associated with better survival, although none were identified as independent prognostic factors.

The CYP4 family consists of several isoforms, including the Cytochrome P450 family 4 subfamily B member 1 (*CYP4B1*), the Cytochrome P450 family 4 subfamily F member 12 (*CYP4F12*), and the Cytochrome P450 family 4 subfamily F member 3 (*CYP4F3*), each with distinct tissue expression patterns and biological functions.

The CYP4B1, due to its ability to activate pro-carcinogens in rodent models, is capable of leading to the formation of DNA adducts and subsequent tumorigenesis; however, the role of CYP4B1 in human cancer is still not well understood. Some studies suggest that CYP4B1 may contribute to the metabolic activation of environmental carcinogens, particularly in tissue such as the lung and bladder, which are common sites of exposure to airborne and waterborne carcinogenic compounds [55].

A study conducted with 20 paired samples of tumor tissue and adjacent normal breast tissue from women diagnosed with invasive ductal carcinoma examined the expression of several cytochrome P450 (CYP) enzymes. The results showed that both the tumor tissue and the surrounding normal breast tissue expressed the enzymes CYP4B1 and others, such as CYP1B1, CYP2B6, CYP2C, CYP2D6, CYP2E1, and CYP11A1 [56].

The CYP4F12 is primarily expressed in the liver and gastrointestinal tract, and is known for its role in metabolizing antihistamines such as ebastine and terfenadine. Additionally, CYP4F12 participates in the oxidation of polyunsaturated fatty acids like arachidonic acid and prostaglandins. The arachidonic acid metabolism generates a variety of bioactive metabolites that are involved in the regulation of inflammation, cell proliferation, and vascular function, which are key processes in cancer development [57,58].

The CYP4F3 isoform exists in two distinct variants that are expressed in different tissues: one form is found in leukocytes, while the other is expressed in the liver. This enzyme plays a crucial role in the metabolism of leukotriene B4 (LTB4), a potent inflammatory mediator that has been implicated in the pathogenesis of various cancers due to its ability to promote cell proliferation, angiogenesis, and resistance to apoptosis, CYP4F3 could potentially modulate inflammatory responses and reduce the tumor-promoting effects of chronic inflammation by inactivating LTB4 [59]. A study demonstrated that the combination of the organophosphorus pesticide parathion and estrogen caused a decrease in the gene expression of CYP4F3 and CYP2F1 enzymes in mammary cells. These results suggest that the presence of estrogen may alter the action of parathion, modifying the metabolism of both xenobiotic and endogenous compounds in mammary tissue [60].

Additionally, CYP4F3 catalyzes the formation of 20-hydroxyeicosatetraenoic acid (20-HETE) from arachidonic acid, a metabolite involved in the regulation of blood pressure, vascular tone, and angiogenesis. Given the importance of angiogenesis in tumor growth and metastasis, alterations in 20-HETE production may influence cancer progression [58,61]. The cytochrome CYP4Z1, associated with breast cancer, contributes to the formation of the signaling molecule 20-HETE [62]. Angiogenesis is inhibited by HET0016, a specific inhibitor of 20-HETE production. 20-HETE has been identified as a second mitogenic messenger of growth factors that induce angiogenesis. The effects of HET0016 on breast cancer cell lines such as MDA-MB-231 were examined in mice and in vitro studies. By lowering the expression of a variety of pro-angiogenic factors, HET0016 decreased the growth of tumors; however, after 21 days, treatment resistance appeared to occur [63]. Estrogen receptor-positive (ER+) breast cancer, which accounts for 80% of occurrences, is the most prevalent subtype of breast cancer and has emerged as a major global public health issue. There is no doubt that ER+ breast cancers are caused by the female hormone estrogen. Although the mechanisms by which estrogen influences breast cancer development have long been understood, more research is needed to fully understand the molecular processes in the ERα signaling pathway that contribute to the progression of ER+ breast cancer [64]. It has been suggested to have a potential role as a tumor-associated antigen since it has been detected in the plasma membrane of MCF-7 cells and the sera of breast cancer patients [65].

Furthermore, its interaction with CYP4Z2P in the miRNA-mediated ceRNET network may inhibit apoptosis while promoting angiogenesis and CDK3 release [66]. Given their multifaceted roles in cancer biology, CYP4 enzymes represent promising therapeutic targets. Inhibitors of specific CYP4 isoforms could be used to modulate the production of pro-inflammatory or metabolites of pro-angiogenesis, thereby limiting tumor growth and improving the efficacy of existing cancer therapies [61]. In a study conducted by Murray G [54], the expression of 21 cytochrome P450 (CYP) enzymes was evaluated in 170 breast tumors. The results showed that CYP4V2, CYP4X1, and CYP4Z1 expression correlated with a higher tumor grade. Additionally, the absence of CYP4V2, as well as CYP2S1, CYP3A4, and CYP26A1, was associated with better survival, although none were identified as independent prognostic factors.

Beyond their roles in xenobiotic metabolism, the enzymes of the CYP4 family have been implicated in broad biological processes relevant to cancer, such as inflammation, angiogenesis, and immune regulation [67]. Chronic inflammation is a well-established risk factor for many types of cancer, as it can create a microenvironment that supports tumor initiation and progression. The metabolites generated by CYP4 enzymes, such as those derived from arachidonic acid, can either promote or resolve inflammation, depending on the specific pathways involved. For example, prostaglandins, which are synthesized from arachidonic acid, can stimulate the inflammatory response and have been shown to promote tumor growth and metastasis in certain cancers [68].

In addition to their involvement in inflammation, CYP4 enzymes play a role in angiogenesis, the formation of new blood vessels from existing vasculature. Angiogenesis is critical for tumor growth, as rapidly proliferating cancer cells require an increased supply of oxygen and nutrients, which is delivered through newly formed blood vessels [69]. Metabolites like 20-HETE, produced by CYP4 enzymes, regulate angiogenic processes, and their dysregulation may contribute to the abnormal vascularization observed in tumors [70]. By influencing both inflammation and angiogenesis, CYP4 enzymes may act as key regulators of the tumor microenvironment, influencing the interactions between cancer cells and their surrounding tissue [71]. Additionally, since CYP4 enzymes are involved in the metabolism of certain chemotherapeutic agents, understanding their activity could help design personalized treatment regimens that minimize drug resistance and enhance therapeutic outcomes [72].

### 3.2. Bioinformatic Analysis of CYPs in Breast Cancer

This study emphasizes *CYP4B1*, *CYP4F12*, and *CYP4F3* gene expression levels, presenting tumor versus normal tissue analysis, their expression adjusted by stages, their correlation with *BRCA1*, *BRCA2,* and *ESR1*, the estrogen receptor status, and the overall survival analysis in breast cancer patients.

#### 3.2.1. Gene Expression Levels in Normal Versus Tumor Tissues

**Figure 1:**
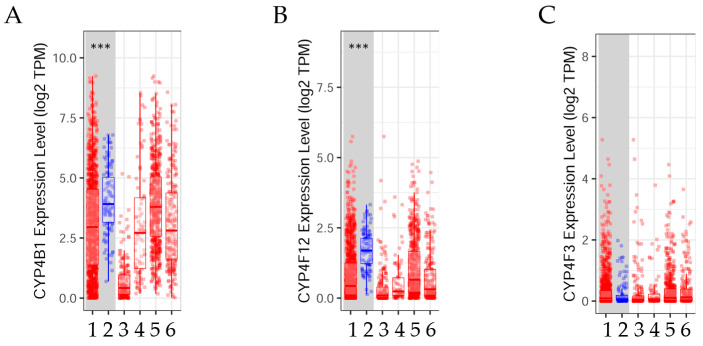
The box plots show the distribution of (**A**) Cytochrome P450 family 4 subfamily B member 1 (*CYP4B1*), (**B**) Cytochrome P450 family 4 subfamily F member 12 (*CYP4F12*), and (**C**) Cytochrome P450 family 4 subfamily F member 3 (*CYP4F3*) gene expression levels in normal (*n* = 112) and tumor (*n* = 1093) tissue in breast cancer; data retrieved from TIMER2.0 dataset [49], http://timer.cistrome.org/, accessed 1 August 2024. Red denotes tumor tissue and blue normal tissue. Wilcoxon test: *** *p* < 0.001. Abbreviations: 1: Breast cancer tumor (*n* = 1093); 2: normal tissue (*n* = 112); 3: basal subtype (*n* = 190); 4: Her2 subtype (*n* = 82); 5: luminal A subtype (*n* = 564); 6: luminal B subtype (*n* = 217).

Gene expression levels between normal and tumor tissue (Figure 1) were estimated using the Tumor Immune Estimation Resource v2.0 (TIMER2.0) in breast cancer [49]. TIMER2.0 is a web server that provides a comprehensive analysis and visualization of the associations between immune infiltrates and genetic or clinical features, and the exploration of cancer-related associations in The Cancer Genome Atlas (TCGA) cohorts through three main components, such as Immune Association, Cancer Exploration, and Immune Estimation. TIMER2.0 GeneDE module of the Cancer Exploration component provided the analysis of tumor and normal tissues.

The results indicated that the expression levels of (A) *CYP4B1* and (B) *CYP4F12* genes were higher (*p* < 0.05) in normal tissue than in tumor tissue, whereas (C) *CYP4F3* expression levels were not significantly different.

#### 3.2.2. *CYP4B1*, *CYP4F12*, and *CYP4F3* Gene Expression Levels Adjusted by Stages

**Figure 2:**
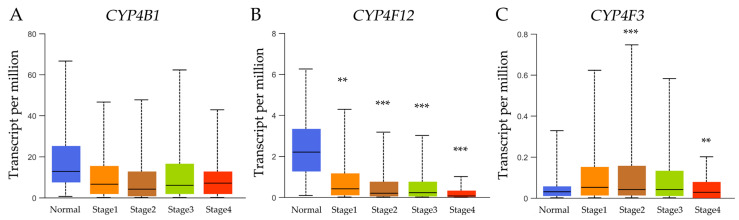
Gene expression levels of (**A**) the cytochrome P450 family 4 subfamily B member 1 (*CYP4B1*), (**B**) the Cytochrome P450 family 4 subfamily F member 12 (*CYP4F12*), and (**C**) the Cytochrome P450 family 4 subfamily F member 3 (*CYP4F3*) across different breast cancer stages, such as stage 1 (*n* = 183), stage 2 (*n* = 615), stage 3 (*n* = 247), and stage 4 (*n* = 20), including normal tissue (*n* = 114) for reference; data provided by UALCAN dataset [50], accessed 12 November 2024. (** *p* < 0.01, *** *p* < 0.001).

The gene expression of CYPs and breast cancer stages was obtained from the University of Alabama at Birmingham Cancer Data Analysis Portal (UALCAN) [50] (https://ualcan.path.uab.edu/), accessed on 12 November 2024 (Figure 2).

According to the breast cancer stage results, no significant expression level of *CYP4B1* can be observed between stages 1–4 and control (normal tissue). However, *CYP4F12* expression levels showed a significant (*p* < 0.01) difference between stage 1 and control and a significant (*p* < 0.001) difference between control and stages 2, 3, and 4. *CYP4F3* expression levels were only significant in stages 2 and 4 (*p* < 0.001 and *p* < 0.01, respectively) in comparison with the control.

#### 3.2.3. Correlation Between *CYP4B1*, *CYP4F12*, and *CYP4F3* and *BRCA1*, *BRCA2*, and *ESR1* Gene Expression Levels in Breast Cancer

**Figure 3:**
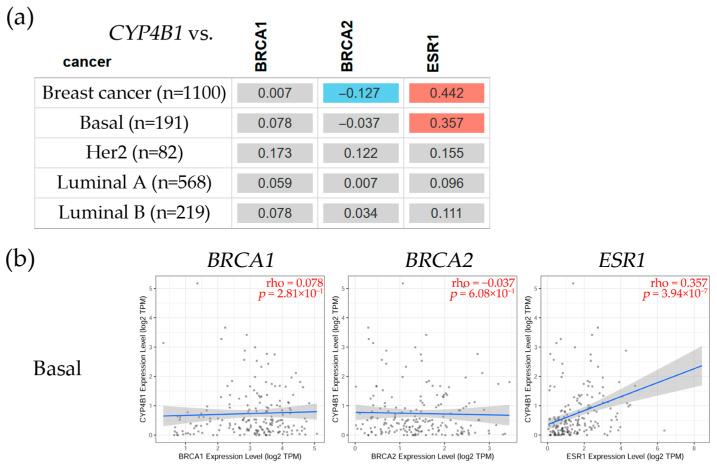
Table (**a**) depicts the cytochrome P450 family 4 subfamily B member 1 (*CYP4B1*) expression levels in several breast cancer subtypes, such as basal, Her2, Luminal A, and Luminal B. Red color shows a significant (Spearman’s, *p* < 0.05) positive correlation, blue denotes a significant (Spearman’s, *p* < 0.05) negative one, and gray indicates a non-significant correlation. (**b**) The scatter plot graphs show the associations between *CYP4B1* and *BRCA1*, *BRCA2*, and *ESR1* gene expression levels in the Basal subtype. These plots depict linear regression lines and correlation coefficients (ρ) for every gene. The linear regression fit is represented by the blue lines that indicate the relationship between *CYP4B1* and *BRCA1*, *BRCA2*, and *ESR1* gene expression levels. The gray area surrounding the regression line represents the confidence interval. The correlation analysis for each box is shown in red in the upper right corner. The statistical significance (Spearman, *p* < 0.05) was determined using the TIMER2.0 web server [49], accessed on 1 August 2024.

To further analyze CYP4s expression, a correlation was observed between *CYP4B1*, *CYP4F12*, *CYP4F3,* and genes with high incidence in breast cancer, such as the BRCA1 DNA repair-associated (*BRCA1*), the BRCA2 DNA repair-associated (*BRCA2*), and the estrogen receptor alpha (*ESR1*), as seen in Figure 3. The analysis was carried out using the TIMER2.0 Gene_Corr module of the Cancer Exploration component [49]. It explores the correlation between a gene of interest and a list of genes in various cancer types, such as breast cancer. A heatmap table displays the partial Spearman’s rho value as the degree of the correlation.

The results showed that this gene had a non-significant correlation with *BRCA1* and *BRCA2,* but it was positively correlated with *ESR1* (ρ = 0.357, *p* = 3.94 × 10^−7^) in the basal subtype.

**Figure 4:**
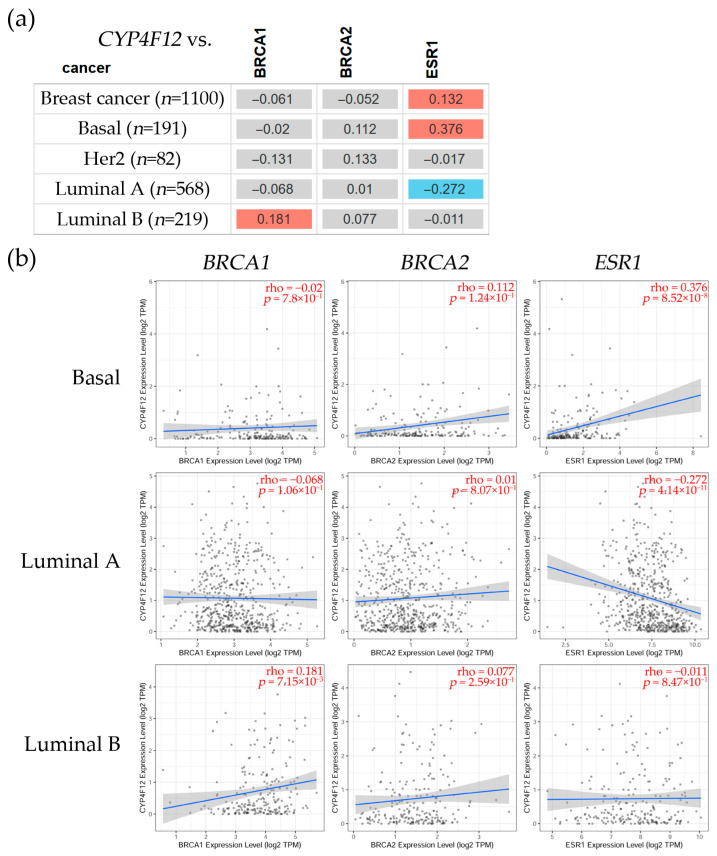
Table (**a**) shows the cytochrome P450 family 4 subfamily F member 12 (*CYP4F12*) expression in several breast cancer subtypes, such as basal, Her2, Luminal A, and Luminal B. Red color shows a significant (Spearman’s, *p* < 0.05) positive correlation, blue denotes a significant (Spearman’s, *p* < 0.05) negative one, and gray indicates a non-significant correlation. (**b**) Scatter plots indicate the associations between *CYP4F12* gene expression and *BRCA1*, *BRCA2*, and *ESR1* expression levels in several breast cancer subtypes. These plots show linear regression lines and correlation coefficients (ρ) for every gene. The linear regression fit is represented by the blue lines, which indicate the relationship between *CYP4F12* expression and *BRCA1*, *BRCA2*, and *ESR1* expression levels, whereas the gray area surrounding the regression line depicts the confidence interval. The correlation analysis for each box is shown in red in the upper right corner. The statistical significance (Spearman, *p* < 0.05) was determined using the TIMER2.0 dataset [49], accessed on 1 August 2024.

The correlation analysis (Figure 4) between *CYP4F12* and *BRCA1*, *BRCA2*, and *ESR1* gene expression indicated that *CYP4F12* had a significant (ρ = 0.181, *p* = 7.15 × 10^−3^) positive correlation with *BRCA1* in the Luminal B subtype; however, it was non-significant with *BRCA2* but positively correlated with *ESR1* (ρ = 0.376, *p* = 8.52 × 10^−8^) in the basal subtype and negatively correlated with ESR1 (ρ = −0.272, *p* = 4.14 × 10^−11^) in the Luminal A subtype.

**Figure 5:**
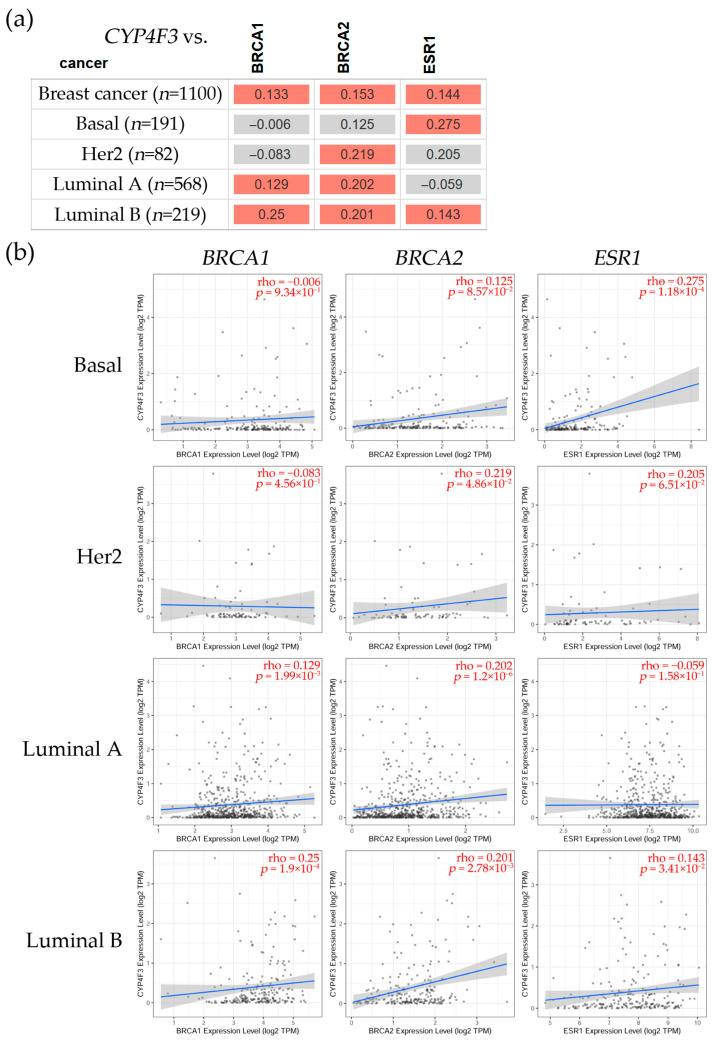
Table (**a**) shows the Cytochrome P450 family 4 subfamily F member 3 (*CYP4F3*) expression in breast cancer subtypes, such as basal, Her2, Luminal A, and Luminal B. Red color shows a significant (Spearman’s, *p* < 0.05) positive correlation and gray indicates a non-significant one. (**b**) Representative scatter plots showing the associations between *CYP4F3* and *BRCA1*, *BRCA2*, and *ESR1* gene expression levels in breast cancer subtypes. These plots depict linear regression lines and correlation coefficients (ρ) for every gene. The linear regression fit is represented by blue lines, which indicate the relationship between *CYP4F3* and *BRCA1*, *BRCA2*, and *ESR1* gene expression levels, whereas the gray area surrounding the regression line depicts the confidence interval. The correlation analysis for each box is shown in red in the upper right corner. The statistical significance (Spearman, *p* < 0.05) was determined using the TIMER2.0 dataset [49], accessed on 1 August 2024.

Figure 5 shows the correlation between *CYP4F3* and BRCA1, *BRCA2*, and *ESR1* gene expression. This indicates that *CYP4F3* had a significant positive correlation with *BRCA1* in Luminal A (ρ = 0.129, *p* = 1.99 × 10^−3^) and Luminal B (ρ = 0.25, *p* = 1.9 × 10^−4^) subtypes. This gene also had a significant positive correlation with *BRCA2* in Her2, Luminal A, and Luminal B subtypes (ρ = 0.219, *p* = 4.86 × 10^−2^, ρ = 0.202, *p* = 1.2 × 10^−6^, and ρ = 0.201, *p* = 2.78 × 10^−3^, respectively). *CYP4F3* had a significant positive correlation with ESR1 expression levels in Basal (ρ = 0.275, *p* = 1.18 × 10^−4^) and Luminal A (ρ = 0.143, *p* = 3.41 × 10^−2^) subtypes.

#### 3.2.4. *CYP4B1*, *CYP4F12*, and *CYP4F3* Gene Expression and the Estrogen Receptor Status

**Figure 6:**
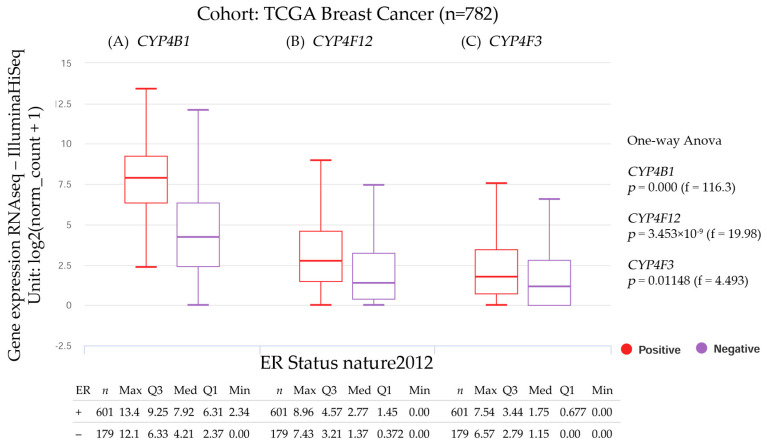
Gene expression and estrogen receptor status of (**A**) Cytochrome P450 family 4 subfamily B member 1 (*CYP4B1*), (**B**) Cytochrome P450 family 4 subfamily F member 12 (*CYP4F12*), and (**C**) Cytochrome P450 family 4 subfamily F member 3 (*CYP4F3*) in breast invasive carcinoma stratified by nature2012. Data were extracted from the University of California, Santa Cruz, UCSC Xena functional genomics explorer [51], accessed on 1 August 2024.

The genes that encode for the CYP proteins and estrogen receptor (ER) status are shown in Figure 6. The results were estimated using the University of California, Santa Cruz (UCSC) Xena functional genomics explorer [51] (https://xena.ucsc.edu/), accessed on 1 August 2024. The statistical significance was computed via a one-way ANOVA test.

The ER status of *CYP4B1*, *CYP4F12*, and *CYP4F3* gene expression levels analyzed in breast cancer patients are shown in Figure 6. *CYP4B1, CYP4F12*, and *CYP4F3* expression levels were higher in patients with a positive ER status than those with a negative ER status. The ER status was analyzed using the UCSC Xena genomic dataset, which included data from the Cancer Genome Atlas (TCGA), International Cancer Genome Consortium (ICGC), and Genomic Data Commons (GDC). The results were mostly positive; the three cases provided a positive significance (*p* < 0.05) in patients with high levels of *CYP4B1*, *CYP4F12*, and *CYP4F3* gene expression. On the other hand, the *CYP4B1* median expression level in patients with positive ER status (7.92) was higher than in patients with negative ER status (4.21). Similarly, *CYP4F12* median expression level was higher in patients with positive ER status (2.77) than in patients with negative ER status (1.37), and CYP4F3 median expression level was also higher in patients with positive ER status (1.75) than in those with negative ER status (1.15).

#### 3.2.5. Overall Survival Analysis of Breast Cancer Patients

**Figure 7:**
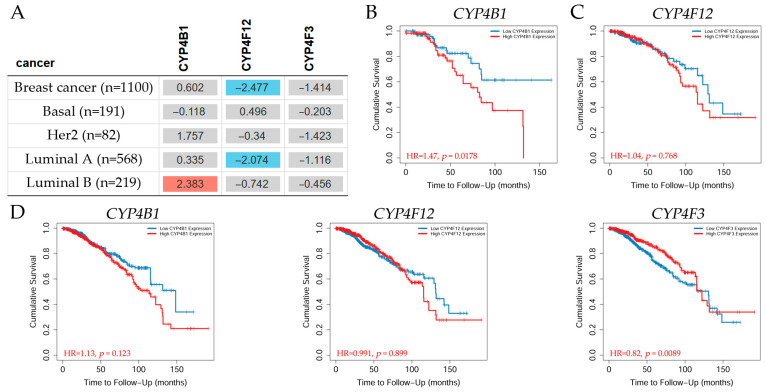
(**A**) Heatmap table depicting the normalized coefficient of the P450 family 4 subfamily B member 1 (*CYP4B1*), the cytochrome P450 family 4 subfamily F member 12 (*CYP4F12*), and the cytochrome P450 family 4 subfamily F member 3 (*CYP4F3*) expression levels in the Cox model, adjusted by clinical stage factor in breast cancer. The red color denotes a statistically significant increased risk (Z-score, *p* < 0.05), the blue color indicates a statistically significant decreased risk (Z-score, *p* < 0.05), and gray denotes a non-significant result. Representative Kaplan–Meier graphs display (**B**) *CYP4B1* in the Luminal B subtype, (**C**) *CYP4F12* in the Luminal A breast cancer subtype, and (**D**) *CYP4B1*, *CYP4F12*, and *CYP4F3* expression levels in all breast cancers. Patients were categorized into high- or low-expression groups. Red indicates high expression levels and blue indicates low expression levels. The group cutoff was set to the median with a cutoff-high and cutoff-low at 50% and a 95% confidence interval. Data retrieved from TIMER2.0 [49] web server, accessed 1 August 2024.

The *CYP4B1*, *CYP4F12*, and *CYP4F3* overall survival (OS) analyses were retrieved using the TIMER2.0 [49] web server in breast cancer. TIMER2.0 Gene_Outcome module assessed the outcome significance of gene expression using the Cox proportional hazard model modified by the clinical stage factor. To display the normalized coefficient of the gene in the Cox model, a heatmap table was created (Figure 7A). The Kaplan–Meier (KM) curves of the genes (Figure 7B–D) were obtained by clicking on a cell in the heatmap when surfing the web server.

The results in the table (Figure 7A) indicate that patients with *CYP4B1* gene expression levels showed a significantly (*p* < 0.05) increased risk of breast cancer in the Lumina B subtype, whereas those patients with *CYP4F12* gene expression levels depicted a significantly (*p* < 0.05) decreased risk of breast cancer in the Luminal A subtype, while *CYP4F3* showed no significant difference. KM analysis indicated that breast cancer patients with high *CYP4B1* expression in the Luminal B subtype (*n* = 219) did not survive within 140 months (Figure 7B), whereas those patients with high *CYP4F12* expression in the Luminal A subtype (*n* = 568) had a decrease in cumulative survival by 70% at about 130 months (Figure 7C); the hazard ratio (HR) values were 1.47 and 1.04, respectively. On the other hand, breast cancer patients (*n* = 1100) with low *CYP4B1* and *CYP4F12* expression levels showed better cumulative survival, whereas patients with high *CYP4F3* expression had better overall survival than their counterparts (Figure 7D).

### 3.3. Adrenocortical Carcinoma

In a study [73], *CYP4B1* expression was markedly suppressed in adrenocortical carcinoma (ACC), with almost complete silencing observed in both adenomas and carcinomas. Quantitative PCR confirmed minimal expression in ACC compared to normal adrenal cortex. Functional assays revealed that the forced expression of *CYP4B1* in ACC cell lines increased cell death and sensitivity to mitotane and cisplatin, suggesting a possible role for *CYP4B1* in tumor suppression and chemosensitization [73].

*CYP4F12* does not exhibit appreciable expression in normal adrenal cortex tissue or adrenocortical carcinoma, according to data from the Human Protein Atlas. To date, there is no published evidence directly implicating this gene in the pathogenesis or clinical behavior of adrenocortical tumors [74]. According to data from the Human Protein Atlas, *CYP4F3* shows no detectable expression in adrenocortical carcinoma, suggesting a limited role in the molecular profile of this malignancy. To date, no specific studies have established a direct association between *CYP4F3* and adrenocortical carcinoma, either as a diagnostic biomarker or a prognostic indicator [74].

### 3.4. Bladder Urothelial Carcinoma

*CYP4B1* and *CYP4F12* display low expression in normal bladder tissue and are detectable in certain cases of bladder urothelial carcinoma (BLCA), as reported by the Human Protein Atlas. Nonetheless, their expression levels are not markedly elevated in this malignancy, and no prognostic, diagnostic, or mechanistic associations have been established to date. Currently, there is a lack of dedicated studies investigating the functional role of *CYP4B1* and *CYP4F12* in the context of BLCA [74]. Elevated RNA expression of cytochrome monooxygenases (*CYP4F11* and *CYP4F3*) and glutathione peroxidase 2 (GPX2), among others, was found in smoking-related malignancies that had minimal baseline leukocyte infiltration. These genes are recognized downstream targets of the redox-sensitive Nrf2 transcription factor pathway and have been connected to the negative regulation of arachidonic acid metabolism, a well-established inflammatory process. However, in BLCA, GPX2 showed a stronger correlation with reduced infiltration of several leukocyte subtypes than Nrf2 markers [75].

### 3.5. Cervical and Endocervical Cancer

*CYP1B1* expression was indicated as a promising diagnostic and prognostic biomarker, as well as a potential therapeutic target in cervical and endocervical cancer (CESC) [76]. In a study conducted by Guoyu Dai, the relationship between gene expression and patient survival was evaluated using Kaplan–Meier survival curves. The results demonstrated that low expression levels of several genes, including *CYP4F12*, were significantly associated with reduced overall survival compared to patients exhibiting high gene expression. These findings suggest a potential protective role of *CYP4F12* in tumor progression and highlight its promise as a prognostic biomarker in specific oncological contexts [77]. Calcitriol treatment was shown to increase *CYP4F3* expression, as confirmed by microarray analysis, qPCR validation, and Western blotting. While the specific role of *CYP4F3* in tumorigenesis remains unclear, its involvement in the metabolism of bioactive lipids and drugs points to it as a promising candidate in cancer research [78].

### 3.6. Colon Adenocarcinoma

The available data indicate that *CYP4B1* expression is very low in colon adenocarcinoma (COAD) tissues. Furthermore, no significant association has been established between its expression and patient prognosis, limiting its usefulness as a biomarker in colon cancer [74]. According to data from the Human Protein Atlas, CYP4F12 displays RNA-level expression in colon cancer tissues, with an average of 8.4 transcripts per million (TPM) observed in COAD samples. However, survival analyses indicate that *CYP4F12* does not serve as a significant prognostic marker in this cancer type [74]. A study explored the role of *CYP4F3* in colon cancer with liver metastasis using transcriptomic datasets from TCGA and GEO. Bioinformatic analyses revealed significant overexpression of *CYP4F3* in metastatic tissues, which was associated with poor overall survival. Additionally, its expression correlated positively with M2 macrophage infiltration, suggesting its involvement in an immunosuppressive tumor microenvironment. miRNA–mRNA network analysis further indicated potential post-transcriptional regulatory mechanisms; collectively, these findings identified *CYP4F3* as a negative prognostic biomarker and a potential therapeutic target in this type of cancer [79].

### 3.7. Esophageal Carcinoma

The CYP4B1 protein was detected in the esophageal mucosa of 25 non-neoplastic human tissues, but its validation via immunoblot was not possible due to the lack of a specific antibody, despite the confirmed presence of its mRNA [80]. A study identified *CYP4F12* as a histone acetylation–responsive gene in esophageal cancer, showing increased expression following trichostatin A treatment through the p300-mediated acetylation of H3K18 and H3K27. Transcriptomic data from TCGA and GSE53624 confirmed its overexpression in tumor tissue and associated high *CYP4F12* levels with a favorable prognosis. Functionally, *CYP4F12* correlated with reduced cell migration, enhanced B-cell infiltration, and downregulation of immune checkpoint markers. These findings positioned *CYP4F12* as a promising prognostic biomarker and potential therapeutic target in esophageal cancer [81]. According to available data from the Human Protein Atlas, CYP4F3 exhibits detectable transcript-level expression in esophageal cancer cell lines. However, the lack of corresponding protein-level evidence limits the interpretation of its clinical significance in this malignancy. Consequently, no prognostic association can currently be established for CYP4F3 in esophageal cancer [74].

### 3.8. Lung Adenocarcinoma

Analysis of the TCGA lung adenocarcinoma (LUAD) cohort (*n* = 567) revealed that *CYP4B1* was significantly overexpressed in tumor tissue compared to normal lung samples (*p* < 0.001), with high expression correlating with improved overall survival (HR = 0.64; *p* = 0.001) [82]. *CYP4B1* expression showed an inverse relationship with myeloid-derived suppressor cells (r = −0.578; *p* < 0.001) and a positive correlation with M2 macrophages (r = 0.211; *p* < 0.001). Additionally, *CYP4B1* was positively associated with immune checkpoints such as PD-1 and PD-L1; these findings suggest that CYP4B1 could serve as a favorable prognostic biomarker and holds predictive value in the context of immunotherapy for lung adenocarcinoma [82]. Data from the Human Protein Atlas show that CYP4F12 had low or undetectable levels of RNA in LUAD and lacked prognostic value; its expression did not correlate with patient survival. Immunohistochemistry records in lung tissue were insufficient to confirm a protein pattern, and so far, no functional studies have been published linking it to the tumor biology of LUAD. Overall, the available evidence indicates that *CYP4F12* is not considered a relevant biomarker or a therapeutic target in lung adenocarcinoma [74].

A meta-analysis of six genome-wide association studies (GWAS) from the TRICL consortium identified *CYP4F3* as a potential gene involved in lung cancer susceptibility. Among 28 significant single-nucleotide polymorphisms (SNPs) identified after FDR correction, 26 were located within *CYP4F3*, with SNP rs4646904 showing a strong association with lung cancer risk (*p* = 8.65 × 10^−6^; FDR = 0.018). This variant may influence RNA splicing and gene expression. Although statistical significance diminished upon inclusion of the Harvard Lung Cancer Study cohort (*p* = 3.52 × 10^−3^), the findings suggested a possible role for *CYP4F3* in lung cancer predisposition, particularly among smokers, warranting further functional investigation [78].

### 3.9. Lung Squamous Cell

An analysis of TCGA-lung squamous cell carcinoma (LUSC) data revealed that elevated *CYP4B1* expression in LUSC correlated with reduced overall survival. Its expression was positively associated with immune infiltrates such as regulatory T cells and M2 macrophages, indicating a potential role in fostering an immunosuppressive tumor microenvironment. These findings suggest that *CYP4B1* could serve as a prognostic and predictive biomarker in LUSC, particularly in the context of immunotherapeutic interventions [83]. Current evidence from the Human Protein Atlas indicates that *CYP4F12* exhibited low or undetectable protein expression levels in LUSC. Immunohistochemical analyses revealed no significant staining in LUSC tissues, suggesting a limited role for this gene in the tumor biology of this cancer subtype [74]. According to the Human Protein Atlas, CYP4F3 exhibited low or undetectable expression at both mRNA and protein levels in LUSC. Although mRNA transcripts were broadly present in these tumors, there was no significant overexpression or reliable protein evidence. Collectively, these data do not indicate a meaningful association between CYP4F3 expression and prognosis in LUSC [74].

### 3.10. Ovarian Serous

In a study of ovarian cancer (OV), *CYP4B1* was identified as a differentially expressed gene in patients with advanced ovarian carcinoma, with significantly lower levels in those who experienced relapse following curative treatment. This reduced expression was associated with an increased risk of recurrence and was consistently validated across multiple platforms, including microarray analysis, NanoString nCounter, and RT-PCR, in both frozen and FFPE tissue samples. As a member of the cytochrome P450 family involved in drug metabolism, decreased *CYP4B1* expression could impair chemotherapeutic efficacy. These findings supported its potential as a prognostic biomarker and therapeutic target in ovarian cancer [84].

A study published in Clinical Cancer Research examined gene expression profiles in the early stages of OV. *CYP4F12* was identified among the genes associated with oxidative stress response, particularly in mucinous tumor subtypes. However, the investigation did not explore the specific functional role or clinical implications of *CYP4F12* in this context [85]. According to the Human Protein Atlas, CYP4F3 exhibits moderate mRNA expression levels, averaging approximately 1.5 TPM, in serous ovarian carcinoma samples. However, no significant correlation between *CYP4F3* expression and patient survival was identified, indicating that *CYP4F3* is unlikely to serve as a prognostic biomarker in this cancer type [74].

### 3.11. Prostate Adenocarcinoma

In a study of prostate adenocarcinoma (PRAD), the expression of *CYP2A6*, *CYP3A5*, and *CYP4B1* genes was evaluated in prostate tissue using real-time quantitative PCR (RT-qPCR). Both normal and cancerous prostate samples were analyzed to assess differential gene expression. The results indicated that *CYP4B1* was expressed in prostate tissue; however, no statistically significant differences were observed between normal and tumor samples. The study concluded that although *CYP4B1* was present in the prostate, its expression levels did not correlate with malignancy, suggesting its limited utility as a biomarker for distinguishing healthy from cancerous prostate tissue in this context [86].

To date, no studies have specifically investigated the expression or functional role of *CYP4F12* in prostate cancer. Data from the Human Protein Atlas indicated low or undetectable expression of this gene in prostate tissue. While polymorphisms in cytochrome P450 family genes were examined concerning prostate cancer, no direct association between *CYP4F12* and disease progression or treatment response was established [74]. Likewise, CYP4F3 exhibited low mRNA expression levels in prostate tissue, with an average of approximately 0.6 TPM. Immunohistochemical analyses further revealed that CYP4F3 protein expression was low or undetectable in prostate tumor samples. Survival analyses using TCGA data indicated that *CYP4F3* lacked prognostic significance in prostate adenocarcinoma [74].

### 3.12. Rectum Adenocarcinoma

The Human Protein Atlas web server indicated that CYP4B1 expression in rectum adenocarcinoma (READ) tissue was low or undetectable, and no significant association was established between its expression levels and patient prognosis [74]. Similarly, CYP4F12 protein expression exhibited low to moderate expression in rectal cancer, with no significant correlation to patient prognosis, according to data from The Human Protein Atlas. Although detectable in tumor tissue, its clinical significance remains undefined [74]. Additionally, CYP4F3 displayed low or nearly undetectable expression at both RNA and protein levels in normal and rectal cancer tissues. No significant overexpression or clear association with prognosis or immune cell infiltration was observed in rectal cancer. Therefore, the evidence does not support a relevant functional role or biomarker potential for CYP4F3 in this malignancy [74].

### 3.13. Stomach Adenocarcinoma

The Human Protein Atlas showed no differential expression pattern or suggested a significant functional role for CYP4B1 in stomach adenocarcinoma (STAD) tissues. No evidence of overexpression or prognostic relevance was reported. Overall, CYP4B1 did not emerge as a notable biomarker or potential therapeutic target in the context of gastric malignancies [74]. Likewise, CYP4F12 exhibited low to undetectable expression in both normal gastric tissue and gastric cancer. No significant overexpression or prognostic association was established. Consequently, based on the evidence, CYP4F12 did not appear to serve as a relevant biomarker or therapeutic target in gastric cancer [74]. Additionally, CYP4F3 showed low to undetectable expression at both mRNA and protein levels in gastric adenocarcinoma according to the Human Protein Atlas, and no significant association was identified between its expression and patient prognosis in this cancer type [74].

### 3.14. Uterine Corpus

CYP4B1 was investigated in various malignancies, including uterine corpus endometrial carcinoma (UCEC), as a potential therapeutic target; however, its role in this type of cancer remained poorly characterized. The Human Protein Atlas indicated low or undetectable expression of CYP4B1 in both normal uterine tissue and endometrial carcinoma, with no significant correlation with patient prognosis. These findings suggest that CYP4B1 has limited diagnostic or therapeutic relevance in uterine cancer [74]. Although direct evidence linking CYP4F12 to uterine cancer is missing, data from the Human Protein Atlas showed its expression across multiple malignancies, including uterine and liver cancers, with higher levels observed in hepatic tumors. In uterine cancer, CYP4F12 was identified as a favorable prognostic marker, suggesting that elevated expression may be associated with improved clinical outcomes [74]. An analysis of the TCGA dataset identified CYP4F3 as a component of a prognostic model in endometrial cancer, where elevated expression correlated with reduced overall survival. Despite the low expression levels in endometrial tissue reported by the Human Protein Atlas, these findings implicate CYP4F3 in tumor progression and warrant further investigation to elucidate its functional role [74].

### 3.15. Bioinformatic Analysis of CYPs in Pan-Cancer

*CYP4B1*, *CYP4F12*, and *CYP4F3* gene expression levels were found to correlate with *ESR1;* then, a tumor versus normal tissue analysis and an overall survival evaluation in several types of cancer were conducted.

#### 3.15.1. Correlation Between *ESR1* and *CYP4B1*, *CYP4F12*, and *CYP4F3* Gene Expression Levels in Several Types of Cancer

**Figure 8:**
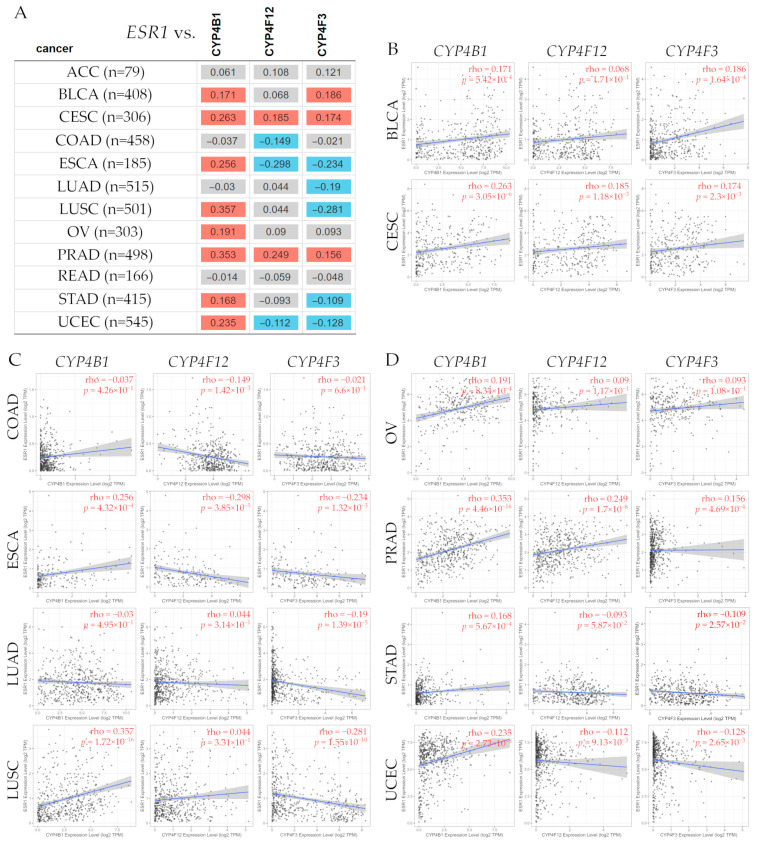
Table (**A**) shows the correlation between estrogen receptor alpha (ESR1) gene expression levels and the cytochrome P450 family 4 subfamily B member 1 (*CYP4B1*), the cytochrome P450 family 4 subfamily F member 12 (*CYP4F12*), and the Cytochrome P450 family 4 subfamily F member 3 (*CYP4F3*) expression in several types of cancer. Red color denotes a significant (Spearman’s, *p* < 0.05) positive correlation, blue denotes a significant (Spearman’s, *p* < 0.05) negative correlation, and gray indicates a non-significant one. (**B**–**D**) Representative scatter plots showing the significant associations between *ESR1* gene expression levels and *CYP4B1*, *CYP4F12*, and *CYP4F3* in several cancer types. These plots depict linear regression lines and correlation coefficients (ρ) for every gene. The linear regression fit is represented by blue lines, which indicate the relationship between the corresponding *CYP* and *ESR1* gene expression levels, while the gray area surrounding the regression line depicts the confidence interval. The correlation analysis for each box is shown in red in the upper right corner. The statistical significance (Spearman, *p* < 0.05) was determined using the TIMER2.0 dataset [49], accessed on 1 August 2024. Abbreviations: ACC: adrenocortical carcinoma; BLCA: bladder urothelial carcinoma; CESC: cervical and endocervical cancer; COAD: colon adenocarcinoma; ESCA: esophageal carcinoma; LUAD: lung adenocarcinoma; LUSC: lung squamous cell carcinoma; OV: ovarian serous cystadenocarcinoma; PRAD: prostate adenocarcinoma; READ: rectum adenocarcinoma; STAD: stomach adenocarcinoma; UCEC: uterine corpus endometrial carcinoma.

To analyze *CYP4B1*, *CYP4F12*, and *CYP4F3* gene expression levels and the estrogen receptor alpha (*ESR1*) gene, a correlation analysis was conducted using the TIMER2.0 web server [49] (http://timer.cistrome.org), accessed on 1 August 2024, as seen in Figure 8.

The correlation analysis between *ESR1* and *CYP4B1*, *CYP4F12*, and *CYP4F3* gene expression levels (Figure 8) indicated that *ESR1* had a significant positive correlation with *CYP4B1* in BLCA (ρ = 0.171, *p* = 5.42 × 10^−4^), CESC (ρ = 0.263, *p* = 3.05 × 10^−6^), ESCA (ρ = 0.256, *p* = 4.32 × 10^−4^), LUSC (ρ = 0.357, *p* = 1.72 × 10^−16^), OV (ρ = 0.191, *p* = 8.34 × 10^−4^), PRAD (ρ = 0.353, *p* = 4.46 × 10^−16^), STAD (ρ = 0.168, *p* = 5.67 × 10^−4^), and UCEC (ρ = 0.235, *p* = 2.73 × 10^−8^). *ESR1* had a positive correlation with *CYP4F12* in CESC (ρ = 0.185, *p* = 1.18 × 10^−3^) and PRAD (ρ = 0.249, *p* = 1.7 × 10^−8^), but a negative correlation in COAD (ρ = −0.149, *p* = 1.42 × 10^−3^), ESCA (ρ = −0.298, *p* = 3.85 × 10^−5^), and UCEC (ρ = −0.112, *p* = 9.13 × 10^−3^). Similarly, *ESR1* was positively correlated with *CYP4F3* in BLCA (ρ = 0.186, *p* = 1.64 × 10^−4^), CESC (ρ = 0.174, *p* = 2.3 × 10^−3^), and PRAD (ρ = 0.156, *p* = 4.96 × 10^−4^), but the correlation was negative in ESCA (ρ = −0.234, *p* = 1.32 × 10^−3^), LUAD (ρ = −0.19, *p* = 1.39 × 10^−5^), LUSC (ρ = −0.281, *p* = 1.55 × 10^−10^), STAD (ρ = −0.109, *p* = 2.57 × 10^−2^), and UCEC (ρ = −0.128, *p* = 2.65 × 10^−3^).

#### 3.15.2. Gene Expression Levels in Tumor Versus Normal Tissues

**Figure 9:**
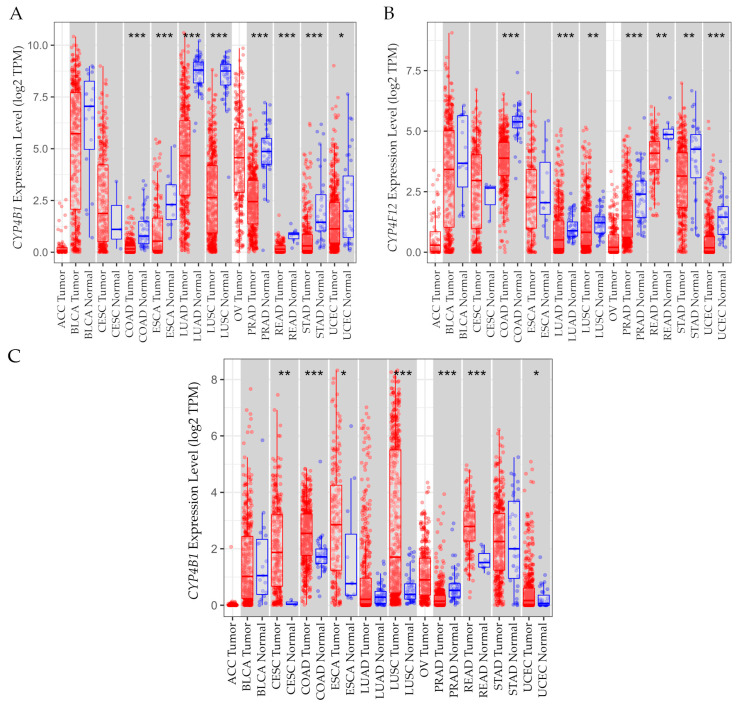
(**A**) Cytochrome P450 family 4 subfamily B member 1 (*CYP4B1*), (**B**) Cytochrome P450 family 4 subfamily F member 12 (*CYP4F12*), and (**C**) Cytochrome P450 family 4 subfamily F member 3 (*CYP4F3*) gene expression levels in normal and tumor tissue in pan cancer; data retrieved from TIMER2.0 dataset [49], http://timer.cistrome.org/, accessed 1 August 2024. Red denotes tumor tissue and blue denotes normal tissue. Wilcoxon test: * *p* < 0.05, ** *p* < 0.01, *** *p* < 0.001. Abbreviations: ACC: adrenocortical carcinoma; BLCA: bladder urothelial carcinoma; CESC: cervical and endocervical cancer; COAD: colon adenocarcinoma; ESCA: esophageal carcinoma; LUAD: lung adenocarcinoma; LUSC: lung squamous cell carcinoma; OV: ovarian serous cystadenocarcinoma; PRAD: prostate adenocarcinoma; READ: rectum adenocarcinoma; STAD: stomach adenocarcinoma; UCEC: uterine corpus endometrial carcinoma.

Gene expression levels between normal and tumor tissue (Figure 9) were estimated using the Tumor Immune Estimation Resource v2.0 (TIMER2.0) in pan-cancer [49].

TIMER2.0 GeneDE module of the Cancer Exploration component was used for the analysis. The results indicated that *CYP4B1* mRNA expression levels were non-significant in ACC, BLCA, CESC, and OV; however, expression showed a statistically significant (either * *p* < 0.05 or *** *p* < 0.001) upregulation in normal tissue compared to tumors in COAD, ESCA, LUAD, LUSC, PRAD, READ, STAD, and UCEC. A significant (either ** *p* < 0.01 or *** *p* < 0.001) upregulation of *CYP4F12* mRNA was observed in normal tissue in comparison with tumors in COAD, LUAD, LUSC, PRAD, READ, STAD, and UCEC, whereas upregulation in ACC, BLCA, CESC, ESCA, and OV was non-significant. A statistically significant (either * *p* < 0.05, or ** *p* < 0.01, or *** *p* < 0.001) *CYP4F3* mRNA overexpression was found in tumors in comparison with normal tissue in CESC, COAD, ESCA, LUSC, READ, and UCEC; however, such overexpression was higher in the normal tissue than tumor tissue in PRAD, whereas ACC, BLCA, LUAD, OV, and STAD showed no significant difference.

#### 3.15.3. Overall Survival Analysis of CYPs in Pan-Cancer

**Figure 10:**
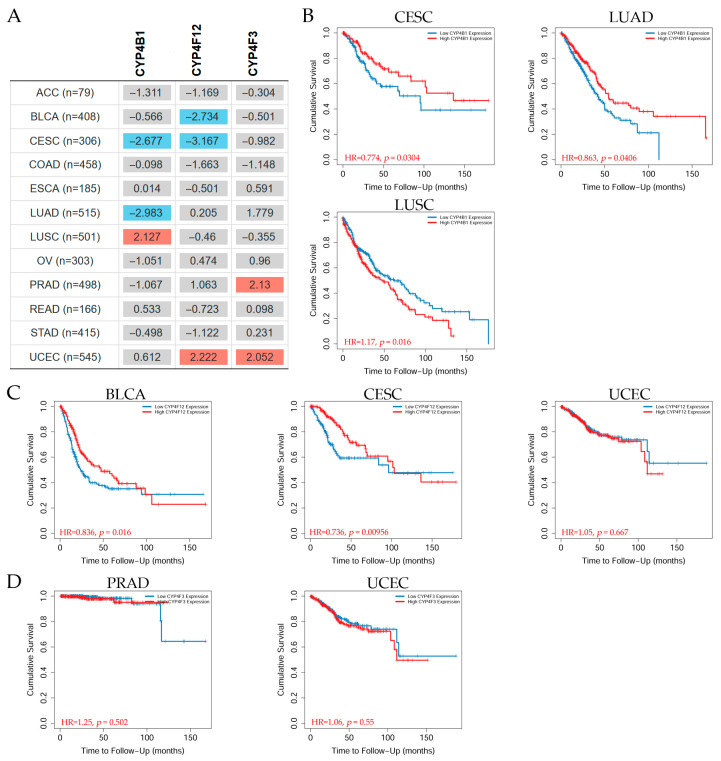
Overall survival in pan-cancer. (**A**) Heatmap table showing the normalized coefficient of P450 family 4 subfamily B member 1 (*CYP4B1*), cytochrome P450 family 4 subfamily F member 12 (*CYP4F12*), and cytochrome P450 family 4 subfamily F member 3 (*CYP4F3*) expression levels in the Cox model, adjusted by clinical factors such as the stage of adrenocortical carcinoma (AAC), bladder urothelial carcinoma (BLCA), cervical and endocervical cancer (CESC), colon adenocarcinoma (COAD), esophageal carcinoma (ESCA), lung adenocarcinoma (LUAD), lung squamous cell carcinoma (LUSC), ovarian serous cystadenocarcinoma, prostate adenocarcinoma (PRAD), rectum adenocarcinoma (READ), stomach adenocarcinoma (STAD), and uterine corpus endometrial carcinoma (UCEC). The red color indicates a statistically significant increased risk (Z-score, p < 0.05), the blue color indicates a statistically significant decreased risk (Z-score, *p* < 0.05), and gray denotes a non-significant result. Representative Kaplan–Meier graphs displaying (**B**) *CYP4B1*, (**C**) *CYP4F12*, and (**D**) *CYP4F3* expression levels in several cancers. Patients were categorized into high- or low-expression groups; red indicates high expression levels and blue indicates low expression levels. The hazard ratio and the log-rank *p*-value are indicated. The group cutoff was set to the median with a cutoff-high and cutoff-low at 50% and a 95% confidence interval. Data were retrieved from the TIMER2.0 dataset [49].

The *CYP4B1*, *CYP4F12*, and *CYP4F3* overall survival (OS) analyses were conducted using the TIMER2.0 [49] in pan-cancer. The TIMER2.0 Gene_Outcome module of the Cancer Exploration component assessed the outcome significance of gene expression using the Cox proportional hazard model, which could be modified by clinical parameters like stage. To display the normalized coefficient of the gene in the Cox model, a heatmap was created (Figure 10A). The Kaplan–Meier (KM) curve of the gene (Figure 10B–D) was obtained by clicking on a cell in the heatmap.

The heatmap table results indicated that patients with *CYP4B1* gene expression levels showed a significantly (*p* < 0.05) decreased risk of CESC and LUAD, but an increased risk of LUSC. Patients with *CYP4F12* expression levels showed a decreased risk of BLCA and CESC, but an increased risk of UCEC. Additionally, those patients with *CYP4F3* gene expression levels depicted an increased risk of PRAD and UCEC. KM analysis showed that patients were grouped into two different categories depending on their CYP4 expression levels: the high-expression (red line) and the low-expression (blue line) groups. Representative KM graphs are presented for (B) *CYP4B1*, (C) *CYP4F12*, and (D) *CYP4F3* gene expression levels. The results in Figure 10B show that patients with low *CYP4B1* expression presented a reduced probability of survival, by 40%, in cervical cancer at about 100 months, lung adenocarcinoma patients did not survive within 120 months, and lung squamous cell carcinoma patients showed reduced survival within 170 months, with HR values were 0.774, 0.863, and 1.17, respectively. Patients with high *CYP4F12* gene expression levels had a decrease in cumulative survival by 70% in bladder cancer at around 120 months, cervical cancer patients with high *CYP4F12* expression showed a decrease in survival by 60% at about 140 months, and endometrial cancer patients had a decrease in survival by 55% at 120 months, HR values were 0.836, 0.736, and 1.05, respectively (Figure 10C). Prostate cancer patients with low *CYP4F3* gene expression showed a decreased cumulative survival of about 35% at about 130 months, whereas endometrial cancer patients with high *CYP4F3* gene expression levels depicted a decreased cumulative survival of about 50% at 120 months.

### 3.16. Advantages of Using CYP Enzymes as Biomarkers in Breast Cancer Compared to Other Solid Cancers

Subtype-Specific Expression: Certain CYP isoforms, like CYP1B1 [87] and CYP2W1 [88], are overexpressed in specific breast cancer subtypes (e.g., basal-like or triple-negative), offering potential for targeted diagnostics and therapies. Hormone Metabolism Insight: CYP enzymes are involved in estrogen metabolism, which is highly relevant in hormone receptor-positive breast cancers [89]. This makes them particularly useful for prognostic and therapeutic decisions in breast cancer [90]. Therapeutic Activation: Some CYPs can activate prodrugs selectively within breast tumors, opening doors for tumor-specific drug delivery with fewer systemic side effects [71,91].

### 3.17. Clinical Trials Based on CYP Family-Based Drugs for Therapeutic Targets in Specific Cancer Settings

There is a growing interest in using CYP enzymes as therapeutic targets in cancer, and several clinical trials have explored this, especially in the context of drug metabolism and resistance [7,39,92,93,94]. One promising approach is gene-directed enzyme prodrug therapy (GDEPT), where genes encoding CYP enzymes are introduced into cancer cells to activate chemotherapy prodrugs directly at the tumor site. This strategy enhances drug efficacy while minimizing systemic toxicity in preclinical and clinical trials. For example, engineered CYP enzymes have been studied for their ability to convert prodrugs into active anticancer agents more efficiently [72]. Additionally, natural CYP inhibitors—like flavonoids—are being investigated for their ability to reduce drug metabolism and improve the bioavailability of chemotherapeutic agents such as paclitaxel. These inhibitors may help overcome drug resistance by suppressing overexpressed CYP enzymes in tumors [89]. Clinical trials have also examined specific CYP isoforms like CYP3A4 in breast cancer settings. For instance, erythromycin, a moderate CYP3A4 inhibitor, was tested for its effect on the pharmacokinetics of palbociclib, a standard breast cancer treatment [95].

### 3.18. CYP1B1 in Tumor-Selective Activation

CYP1B1 is often overexpressed in tumors but not in normal tissues. This makes it a prime target for tumor-selective prodrugs [96]. For example, compounds like aminoflavone and Phortress are activated by CYP1B1, releasing cytotoxic agents only in cancer cells [97]. Hormone-Dependent Cancers CYP enzymes like CYP19 (aromatase) and CYP17 have been successfully targeted in breast and prostate cancers, respectively [98,99]. Aromatase inhibitors (e.g., anastrozole) are already standard in estrogen-receptor-positive breast cancer therapy [100,101,102]. The NIH ClinicalTrials.gov database provides comprehensive access to both ongoing and completed clinical trials, systematically organized by CYP isoform, cancer subtype, and therapeutic modality. Notably, several investigations involving CYP2D6 polymorphisms have contributed to the refinement of tamoxifen-based therapy in breast cancer patients, enabling a pharmacogenomically guided, individualized treatment strategy [103,104,105].

Aromatase inhibitors such as anastrozole and letrozole are now frontline treatments for ER+ breast cancer, effectively reducing estrogen synthesis in postmenopausal women [102,106,107,108,109]. Some key references that support the use of CYP enzymes in hormone-dependent cancers are as follows:

Aromatase (CYP19) and breast cancer: Aromatase is a cytochrome P450 enzyme that catalyzes the conversion of androgens to estrogens [110,111,112].

CYP17 and Prostate Cancer: CYP17 is crucial in androgen biosynthesis [113]. Inhibitors like abiraterone acetate (Zytiga) are FDA-approved for treating castration-resistant prostate cancer [99,114].

CYP2D6 and Tamoxifen Metabolism: CYP2D6 polymorphisms significantly affect tamoxifen metabolism and therapeutic response in breast cancer [103,105]. This is a key area of pharmacogenomic research [115].

Clinical Trials Using Anastrozole (CYP19 Inhibitor). The National Cancer Institute (NCI) lists multiple ongoing trials evaluating anastrozole in breast cancer. These include the following:

The DEBRA Trial: Testing radiation plus hormonal therapy vs. hormonal therapy alone in early-stage breast cancer [116,117,118].

LoTam Trial: Investigating low-dose tamoxifen for invasive breast cancer [119,120].

CAMBRIA-2 Study: Evaluating camizestrant vs. standard endocrine therapy in ER+/HER2- early breast cancer [121,122].

Some studies [123,124] discussed how CYP2D6 polymorphisms affected tamoxifen metabolism. While CYP2D6 genotyping can predict endoxifen levels [125], a comprehensive review in The Oncologist concluded that aromatase inhibitors were equivalent or superior to tamoxifen as first-line therapy for metastatic breast cancer and as neoadjuvant treatment [126]. They also offer a more favorable toxicity profile in many cases [127].

## 4. Conclusions

Overall, the document highlights the complex relationship between CYP enzymes and estrogen receptors in breast cancer, suggesting avenues for further research and potential therapeutic interventions. The study provides a comprehensive approach to the expression, clinical relevance, and genetic variation of the *CYP4B1*, *CYP4F12*, and *CYP4F3* genes in breast and other cancers. The detected differential expression patterns between normal and tumor tissue suggest a functional role in tumorigenesis. Additionally, the positive correlation of these genes with estrogen receptor status and clinical data strengthen their potential as predictive biomarkers. Overall, the article emphasizes the functional importance of CYP4, highlighting the complex interplay between CYP enzymes and estrogen receptors in breast cancer. This study demonstrates their relevance not only in chemical detoxification but also in fundamental physiological processes such as hormonal regulation and oxidative stress response, indicating new avenues for future research and potential therapeutic interventions. Several cancer types beyond breast cancer were also explored, revealing multiple gene functions depending on the tissue context. While expression of these genes was generally low or undetectable in many tissues, specific organ-related contexts revealed functional and prognostic implications. Based on clinical prognostic relevance, *CYP4B1* showed significant activity in adrenocortical cancer as a tumor-suppressor with drug-sensitizing effects; in lung adenocarcinoma, it was associated with improved survival, whereas in lung squamous cell carcinoma, it correlated with poor prognosis and an immunosuppressive profile; in ovarian cancer, it was linked to recurrence and chemoresistance. Significant associations of *CYP4F12* included favorable prognosis in cervical cancer; in esophageal cancer, they were associated with reduced migration and immune modulation; and in ovarian mucinous tumors, they were associated with responsiveness to oxidative stress. In the case of *CYP4F3*, the findings included its emergence as a progression marker in bladder cancer, modulated by calcitriol in cervical cancer; it was also associated with poor prognosis in colorectal liver metastasis and included in a prognostic model of endometrial cancer. Collectively, these findings suggested the potential functional significance of these genes as predictive biomarkers or emerging therapeutic targets in selected cancer types. On the other hand, even though the mechanisms by which estrogen influences breast cancer development have long been understood, more research is needed to fully understand the molecular processes in the 17ß-estradiol-estrogen receptor α (ERα) signaling pathway that contribute to the progression of ER+ breast cancer, particularly lipid metabolism, to provide more options for customized and individualized therapy.

## Data Availability

The data presented in this study are openly available in the Tumor Immune Estimation Resource v2.0 (TIMER2.0) [49], freely available at http://timer.cistrome.org (accessed on 1 August 2024); The University of Alabama at Birmingham Cancer Data Analysis Portal (UALCAN), available at https://ualcan.path.uab.edu/ [50] (accessed on 12 November 2024); the University of California, Santa Cruz, UCSC Xena functional genomics explorer [51], freely available at https://xena.ucsc.edu/, accessed on 1 August 2024. The data generated in the present study may be requested from the corresponding author.

## Abbreviations

The following abbreviations are used in this manuscript:

ER-	Estrogen receptor-negative
ER+	Estrogen receptor-positive
CYP	Cytochrome P450
CYP4B1	Cytochrome P450 family 4 subfamily B member 1
CYP4F12	Cytochrome P450 family 4 subfamily F member 12
CYP4F3	Cytochrome P450 family 4 subfamily F member 3
LTB4	Leukotriene B4
20-HETE	20-hydroxyeicosatetraenoic acid
TCGA	The Cancer Genome Atlas
GTEx	Genotype-tissue expression
GEPIA2	Gene Expression Profiling Interactive Analysis
UALCAN	University of Alabama at Birmingham Cancer Data Analysis Portal
HPA	Human Protein Atlas
BRCA1	BRCA1 DNA repair-associated
BRCA2	BRCA2 DNA repair-associated
ESR1	Estrogen receptor alpha
TIMER2.0	Tumor Immune Estimation Resource v2.0
ρ	Correlation coefficients
UCSC Xena	University of California, Santa Cruz, Xena explorer
ICGC	International Cancer Genome Consortium
GDC	Genomic Data Commons
OS	Overall survival
KM	Kaplan–Meier
